# A photoactivatable tumor‐targeting in situ nanovaccine for large‐volume tumor therapy

**DOI:** 10.1002/smo2.70014

**Published:** 2025-08-19

**Authors:** Junying Ding, Xueze Zhao, Saran Long, Wen Sun, Jianjun Du, Jiangli Fan, Xiaojun Peng

**Affiliations:** ^1^ State Key Laboratory of Fine Chemicals Frontiers Science Center for Smart Materials Dalian University of Technology Dalian China; ^2^ Department of Chemistry The University of Hong Kong Hong Kong China; ^3^ Ningbo Institute of Dalian University of Technology Ningbo China; ^4^ State Key Laboratory of Fine Chemicals College of Materials Science and Engineering Shenzhen University Shenzhen China

**Keywords:** in situ nanovaccine, large‐volume tumor, photoactivatable, photodynamic immunotherapy, tumor‐targeting

## Abstract

The clinical application of tumor vaccines is hindered by challenges such as time‐consuming and costly production processes. In this context, in situ cancer vaccines represent a promising strategy by leveraging endogenous tumor antigens to elicit robust antitumor T cell responses. Herein, a photoactivatable tumor‐targeting in situ nanovaccine, Lipo‐D8‐6, was constructed using cRGD‐functionalized liposomes that co‐encapsulated the photosensitizer chlorin e6 and a cleavable immunoadjuvant conjugate D8, allowing light‐triggered synchronous activation of three therapeutic modules. Upon near‐infrared light irradiation, Lipo‐D8‐6 generates reactive oxygen species that exert direct cytotoxicity on tumor cells and induce immunogenic cell death, while concurrently cleaving the responsive linker within D8 to achieve the controlled release of R848. In vivo biodistribution analysis confirmed the superior intratumoral accumulation of Lipo‐D8‐6, facilitating precise treatment. In a large‐volume tumor model, the nanovaccine exhibited pronounced antitumor efficacy, accompanied by enhanced tumor infiltration of CD8^+^ T cells. Overall, this work provides a simplified and effective approach for developing in situ nanovaccines that enable synergistic photodynamic immunotherapy with precise spatiotemporal control over immune activation.

## Graphical abstract

This study presents a photoactivatable tumor‐targeting in situ nanovaccine Lipo‐D8‐6, which generates ROS to synchronously activate three therapeutic modules, including direct tumor killing effect, ICD induction, and the controlled release of immunoadjuvant, thereby promoting DC maturation. In vivo analysis demonstrates that Lipo‐D8‐6 exhibits high intratumoral accumulation and significantly inhibits large‐volume tumor growth with enhanced CD8^+^ T cell tumor infiltration.

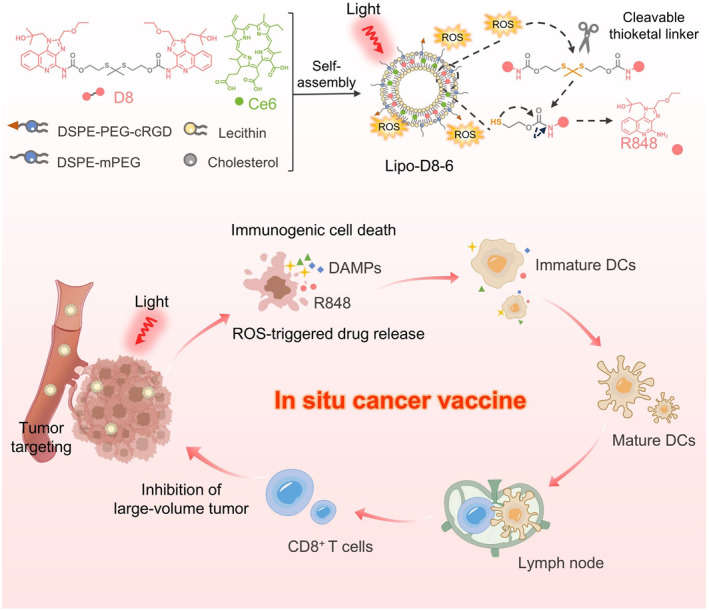

## 1 INTRODUCTION

Tumor vaccines, designed to enhance tumor‐specific T cell responses,^[1]^ have long been regarded as a key tool in cancer immunotherapy.^[2]^ Despite significant advancements, their clinical application remains limited by several challenges, including tumor heterogeneity,^[3]^ the complexity of neoantigen identification,^[4]^ and the time‐consuming and costly manufacturing process required for personalized neoantigen vaccines.[^4^, ^5^] In recent years, in situ tumor vaccines have emerged as a therapeutic alternative by utilizing endogenous antigens generated at the tumor site to trigger immune activation, thereby eliminating the need for antigen identification and delivery.^[6]^

Photodynamic therapy (PDT) has attracted considerable attention as an effective cancer treatment approach due to its high spatiotemporal precision and minimal invasiveness.^[7]^ Its therapeutic mechanism involves photosensitizers that, upon light irradiation, sensitize molecular oxygen to generate reactive oxygen species (ROS).^[8]^ In addition to direct cytotoxicity, PDT exerts immunomodulatory effects by triggering immunogenic cell death (ICD) of tumor cells.^[9]^ The ability of cancer therapies to induce ICD is clinically significant and may serve as an in situ vaccine.[^6^, ^10^] During this process, dying tumor cells release damage‐associated molecular patterns (DAMPs), which initiate a cascade of immune responses.^[11]^

Accumulating evidence suggests that the strategic incorporation of immunoadjuvants can amplify both the strength and speed of vaccine‐induced immunity.^[12]^ For example, the Toll‐like receptor 7/8 (TLR7/8) agonist resiquimod (R848) has been shown to promote dendritic cell (DC) maturation and CD8^+^ T cell priming.^[13]^ Therefore, the combination of TLR agonists with PDT capable of inducing ICD offers a promising approach to activate robust systemic immunity, with the potential to address challenging lesions, such as large‐volume tumors. However, two major barriers limit this combination approach. First, the insufficient tumor accumulation of traditional photosensitizers restricts the efficiency of ROS generation.^[14]^ Second, the poor pharmacokinetic profile of small molecule TLR agonists, along with the potential toxicity of systemic distribution, presents significant challenges to their therapeutic application.^[15]^

**FIGURE 1:**
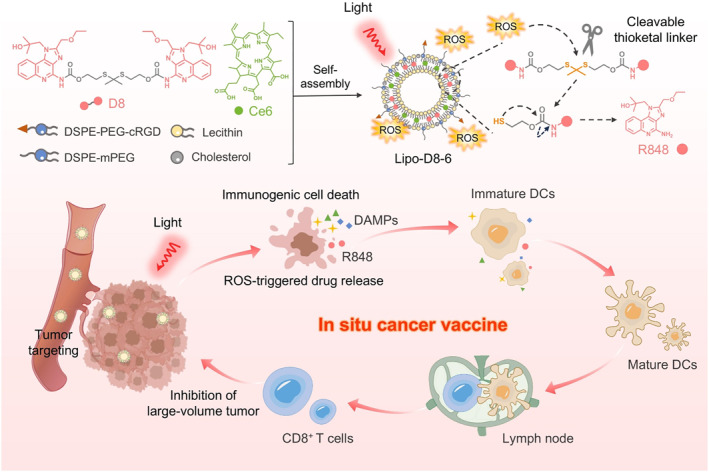
Schematic illustration of the photoactivatable tumor‐targeting in situ nanovaccine Lipo‐D8‐6.

To overcome these challenges, this study developed an in situ nanovaccine (Lipo‐D8‐6) based on cRGD‐functionalized liposomes co‐encapsulating the photosensitizer chlorin e6 (Ce6) and a cleavable immunoadjuvant conjugate D8, as illustrated in Figure 1. This nanovaccine achieves dual optimization of tumor‐specific delivery and stimuli‐responsive drug release, thereby enhancing antitumor immunity while ensuring favorable biosafety. Upon near‐infrared (NIR) light irradiation, Lipo‐D8‐6 generates ROS, leading to direct cytotoxicity and ICD, along with the exposure of DAMPs. Concurrently, ROS‐mediated cleavage of the responsive linker facilitates the controlled release of R848. This coordinated system enhances DC maturation. In vivo biodistribution analysis demonstrated substantial tumor accumulation of Lipo‐D8‐6 following intravenous administration. In the large‐volume tumor model, the nanovaccine significantly inhibited tumor growth and promoted CD8^+^ T cell tumor infiltration.

## 2 RESULTS AND DISCUSSION

### 2.1 Synthesis and characterization of lipo‐D8‐6

**FIGURE 2:**
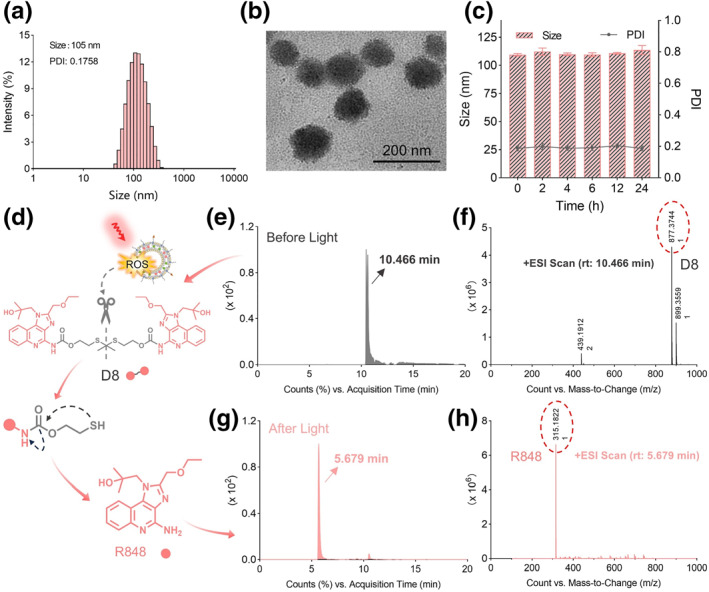
(a) The size distribution of Lipo‐D8‐6. (b) The representative transmission electron microscopy image of Lipo‐D8‐6. (c) Stability assay of Lipo‐D8‐6 after incubation with 10% (v/v) fetal bovine serum. (d) Schematic illustration of the responsive behavior of Lipo‐D8‐6. (e) HPLC chromatograms of Lipo‐D8‐6 without light irradiation. (f) The mass spectrometry corresponding to Figure 2e. (g) HPLC chromatograms of Lipo‐D8‐6 with 660 nm light irradiation. (h) The mass spectrometry corresponding to Figure 2g. HPLC, high performance liquid chromatography.

To construct the Lipo‐D8‐6 nanovaccine, the cleavable immunoadjuvant conjugate D8 was first synthesized and characterized using mass spectrometry (MS) and nuclear magnetic resonance (NMR) spectroscopy (Figures S1−S5). Liposomes were selected as the nanocarrier platform due to their high biocompatibility, favorable pharmacokinetic properties, and versatile drug‐loading capacity.^[16]^ Subsequently, Ce6 and D8 were encapsulated within tumor‐targeting liposomes to form Lipo‐D8‐6. As shown in Figure 2a, dynamic light scattering (DLS) analysis revealed that Lipo‐D8‐6 nanoparticles exhibited a hydrodynamic diameter of 105 nm with a polydispersity index (PDI) of 0.1758, indicating a narrow and unimodal size distribution. Transmission electron microscopy (TEM) further confirmed the spherical shape of Lipo‐D8‐6 with uniform size and regular morphology (Figure 2b). To evaluate serum stability, the particle size distribution was monitored during 24 h incubation with 10% fetal bovine serum (FBS). Only slight fluctuations in size and PDI were observed (Figure 2c), indicating the excellent stability of Lipo‐D8‐6.

Then, to investigate the photoactivatable drug‐release behavior of Lipo‐D8‐6 (Figure 2d), high performance liquid chromatography (HPLC) was employed. Upon exposure to 660 nm light (50 mW/cm^2^), the release of R848 from Lipo‐D8‐6 increased over time, reaching a cumulative release of over 80% under continuous irradiation (Figure S6). Moreover, HPLC‐MS analysis showed that prior to light exposure, a chromatographic peak appeared at 10.466 min exhibited a mass‐to‐charge ratio (*m/z*) of 877.3744 (Figure 2e,f), corresponding to the protonated form ([M+H]^+^) of compound D8. After light irradiation, a chromatographic peak was detected at 5.679 min (Figure 2g), and MS confirmed this signal as the protonated form ([M+H]^+^) of R848 (Figure 2h), with the measured *m/z* value closely matching the calculated value. These results provide evidence for the successful light‐triggered release of R848 and demonstrate the potential of Lipo‐D8‐6 as a photoactivatable drug delivery system.

### 2.2 Cell uptake and antitumor efficacy in vitro

**FIGURE 3:**
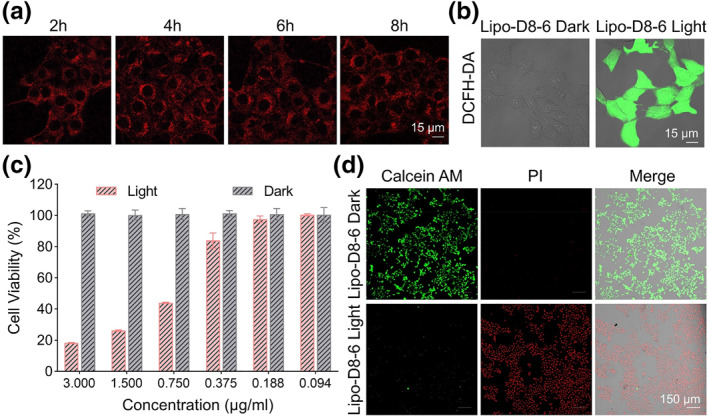
(a) The cell uptake of Lipo‐D8‐6. (b) reactive oxygen species generation detection of Lipo‐D8‐6 in 4T1 cells with DCFH‐DA. (c) Cell viability of 4T1 cells treated with Lipo‐D8‐6 at different concentrations under dark and light irradiation. (d) Live/death cells imaging of Lipo‐D8‐6 with or without light irradiation.

The cellular uptake of Lipo‐D8‐6 in 4T1 cells was evaluated using confocal laser scanning microscopy (CLSM). As illustrated in Figure 3a and Figure S7, 4T1 cells exhibited a time‐dependent uptake, with fluorescence signals reaching the maximum level within 4 h. The ROS generation capability of Lipo‐D8‐6 was subsequently investigated using DCFH‐DA as an intracellular ROS indicator.^[17]^ As shown in Figure 3b, no detectable green fluorescence was observed in cells treated with Lipo‐D8‐6 under dark condition, indicating the absence of ROS generation. In contrast, cells in the Lipo‐D8‐6 Light group displayed strong green fluorescence, attributed to the oxidation product DCF, confirming the light‐triggered ROS generation mechanism for cancer cell eradication.

To further evaluate the antitumor efficacy, the cytotoxicity of Lipo‐D8‐6 was determined by MTT assay. As illustrated in Figure 3c, Lipo‐D8‐6 exhibited concentration‐dependent cytotoxicity under 660 nm light irradiation, while minimal toxicity was observed under dark conditions. Additionally, calcein acetoxymethyl ester (Calcein AM) and propidium iodide (PI) co‐staining was performed to visually distinguish live cells (green fluorescence) from dead cells (red fluorescence), providing a more intuitive assessment of phototoxicity. Consistent with the MTT results, CLSM images of the PDT‐treated group showed predominant red fluorescence, confirming the potent light‐induced cytotoxicity of Lipo‐D8‐6 against cancer cells (Figure 3d). In contrast, cells in the non‐irradiated group exhibited only green fluorescence, indicating the favorable biosafety of this nanovaccine in the absence of light activation.

### 2.3 ICD cascade induction and dendritic cells stimulation in vitro

The process of ICD in tumor cells is characterized by the release of DAMPs, including calreticulin (CRT) exposure on the cell membrane, extracellular secretion of high mobility group box 1 (HMGB1) and adenosine triphosphate (ATP) efflux.[^11^, ^18^] These biomarkers collectively contribute to the establishment of an immunostimulatory microenvironment, promoting DC maturation and subsequent T cell‐mediated antitumor responses.^[19]^

**FIGURE 4:**
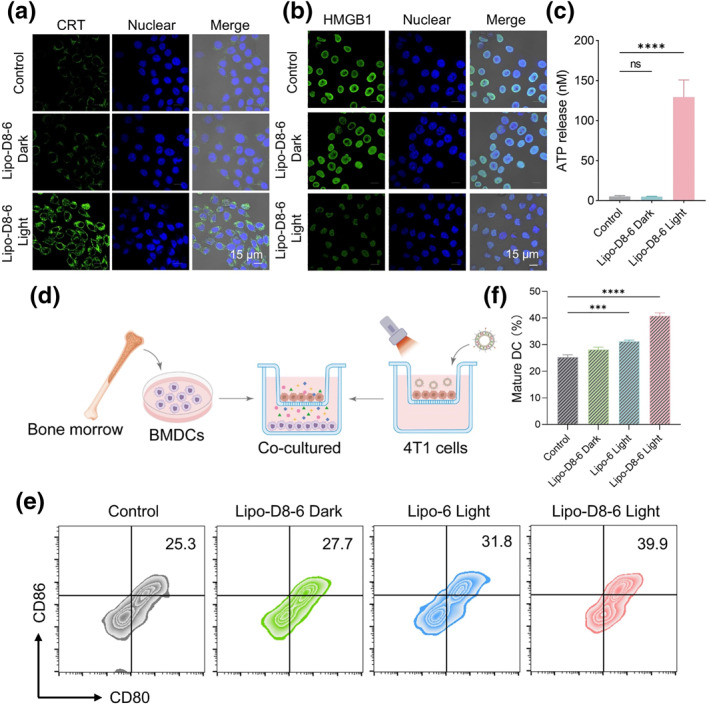
(a) CLSM images of calreticulin after different treatments. (b) CLSM images of high mobility group box 1 after different treatments. (c) Extracellular adenosine triphosphate levels after different treatments. (d) Schematic illustration of BMDCs stimulation with a transwell system. (e) Representative flow cytometry plots and (f) statistical analysis of BMDCs maturation after different treatments. BMDCs, bone marrow‐derived dendritic cells; CLSM, confocal laser scanning microscopy.

To evaluate the ICD‐inducing capacity of Lipo‐D8‐6 in tumor cells, immunofluorescence staining was used to assess the surface translocation of CRT and the nuclear release of HMGB1 following various treatment conditions. As shown in Figure 4a and Figure S8, distinct green fluorescence was detected on the cell membrane of tumor cells treated with Lipo‐D8‐6 under light irradiation, indicating CRT exposure after PDT treatment. In contrast, negligible fluorescence signals were observed in both the control and Lipo‐D8‐6 Dark groups. Additionally, the Lipo‐D8‐6 Light group exhibited a marked reduction in nuclear HMGB1 fluorescence and a significant increase in extracellular ATP levels (Figure 4b,c and Figure S9). This tripartite response confirms that Lipo‐D8‐6 is capable of eliciting ICD.

As key antigen‐presenting cells, DCs play a crucial role in initiating antitumor immune responses. To further evaluate the effect of Lipo‐D8‐6–mediated photodynamic immunotherapy on DC maturation, bone marrow‐derived dendritic cells (BMDCs) were cocultured with 4T1 cells subjected to different treatments in a transwell system for 24 h (Figure 4d). As shown in Figure 4e,f, the Lipo‐D8‐6 Light group exhibited the highest proportion of CD80^+^ CD86^+^ mature BMDCs (39.9%) among all groups. These findings suggest that this combinatorial approach holds strong potential to elicit a robust immune response against tumors.

### 2.4 In vivo fluorescence imaging of lipo‐D8‐6

**FIGURE 5:**
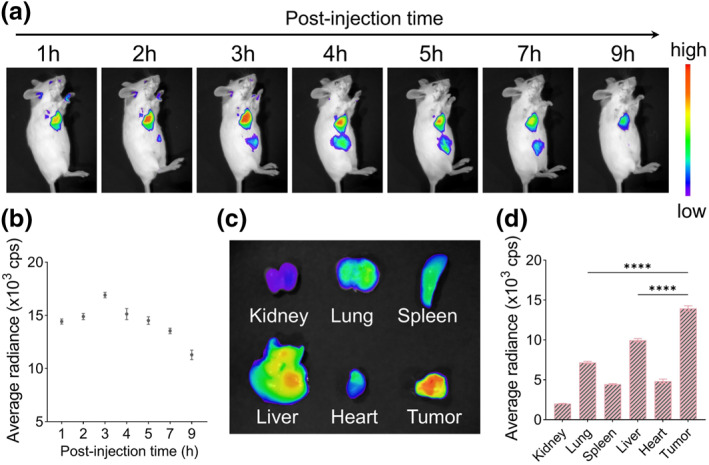
(a) In vivo fluorescence imaging of tumor‐bearing mice at a series of time points after intravenous injection of Lipo‐D8‐6. (b) Average radiance in tumor sites at different time points. (c) Ex vivo fluorescence imaging of main organs and tumor. (d) Average radiance in main organs and tumor.

Given the promising therapeutic efficacy of Lipo‐D8‐6 observed in vitro, orthotopic 4T1 breast tumor‐bearing Balb/c mice were used to evaluate its antitumor performance in vivo. First, the biodistribution of Lipo‐D8‐6 was investigated by fluorescence imaging following intravenous injection. As illustrated in Figure 5a,b, Lipo‐D8‐6 progressively accumulated in tumor tissue, reaching peak intensity at 3 h post‐injection. Comparative analysis of fluorescence intensity between tumor and major organs (heart, liver, spleen, lung, and kidneys) revealed pronounced tumor‐specific accumulation (Figure 5c), with fluorescence intensity in tumor tissue being 1.94‐fold and 1.4‐fold higher than that of the lung and liver, respectively (Figure 5d). This biodistribution profile indicated the superior tumor‐targeting capability of Lipo‐D8‐6, allowing localized treatment confined to the tumor site and thereby eliciting an in situ vaccine effect. These advantages lay a solid foundation for precise in vivo photodynamic immunotherapy.

### 2.5 In vivo antitumor efficacy of lipo‐D8‐6

**FIGURE 6:**
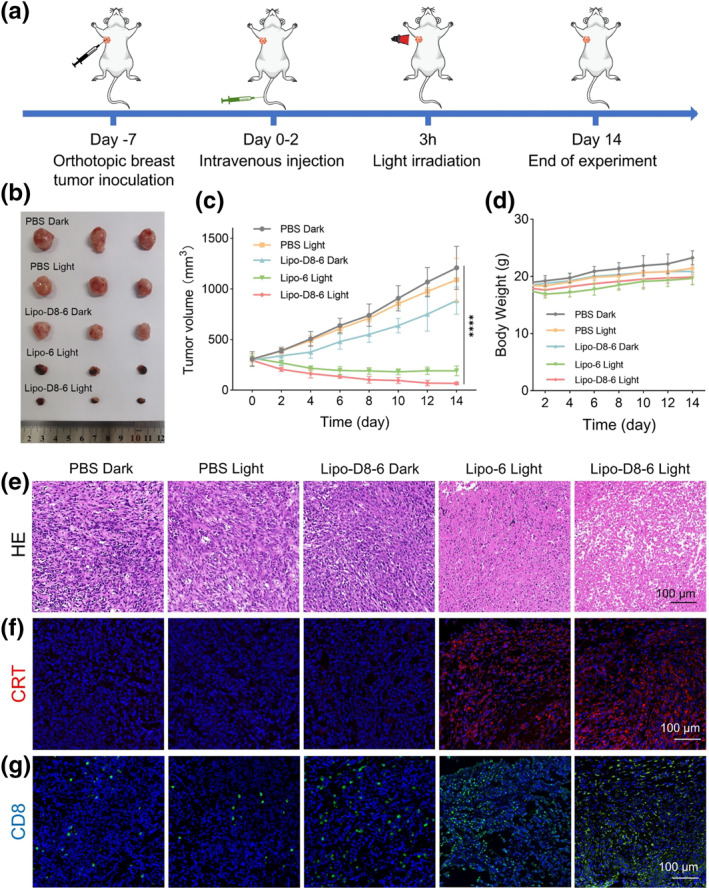
(a) Illustration of the treatment schedule for evaluating antitumor effects on the large‐volume tumor model. (b) Photographs of excised tumors in different treatment groups on day 14. (c) Tumor volume growth curves after different treatments. (d) Body weight of mice in different treatment groups. (e) H&E staining images of tumor sections after different treatments. (f) Immunofluorescence staining of calreticulin in tumor tissue sections from different treatment groups. (g) Immunofluorescence staining of CD8^+^ T cells in tumor tissue sections from different treatment groups.

Subsequently, the therapeutic efficacy of Lipo‐D8‐6 was evaluated in a large‐volume orthotopic breast tumor model (initial volume ≈ 300 mm^3^) (Figure 6a). As shown in Figure 6b,c and Figure S10, rapid tumor progression was observed in the PBS Dark, PBS Light, and Lipo‐D8‐6 Dark groups. In contrast, tumor suppression occurred in the light‐irradiated treatment groups. Notably, the Lipo‐D8‐6 Light group exhibited the most significant tumor inhibition, demonstrating that the combination of PDT and an immunoadjuvant enhances therapeutic efficacy. Tumor tissue morphology across different treatment groups was evaluated using hematoxylin and eosin (H&E) staining. As illustrated in Figure 6e, tumor sections from both PBS groups showed densely packed cellular architecture with intact nuclear morphology, indicative of viable tumor cells. In contrast, tumor sections from the Lipo‐D8‐6 Light group exhibited severe tumor cell death with loss of morphological integrity, providing conclusive evidence that Lipo‐D8‐6 exerts cytotoxic effects under light irradiation. Moreover, the body weight of mice remained stable throughout the treatment period (Figure 6d), and H&E staining of major organs revealed no obvious abnormalities at the end of treatment (Figure S11), indicating that Lipo‐D8‐6 possesses favorable in vivo biosafety without causing noticeable side effects.

To elucidate the mechanism underlying the superior therapeutic efficacy, immunofluorescence staining was performed to evaluate CRT exposure and CD8^+^ T cell infiltration in tumors from different treatment groups. As shown in Figure 6f,g, the levels of both markers were significantly upregulated in the Lipo‐D8‐6 Light group compared to the other groups. These results suggest that Lipo‐D8‐6 upon light irradiation functions as an in situ vaccine to initiate antitumor immunity.

## 3 CONCLUSION

To construct an in situ vaccine with high tumor specificity, cRGD‐functionalized liposomes were employed to co‐deliver the photosensitizer Ce6 and the immunoadjuvant conjugate D8, forming the nanovaccine Lipo‐D8‐6 for synergistic therapy. Upon NIR light irradiation, Lipo‐D8‐6 generates ROS that directly kill tumor cells, induce ICD accompanied by the exposure of DAMPs, and trigger the cleavage of the responsive linker to enable the controlled release of R848, thereby promoting DCs maturation. In vivo fluorescence imaging demonstrated that, following intravenous injection, Lipo‐D8‐6 achieved the highest accumulation in tumor tissues compared to other major organs. In the large‐volume tumor model, this nanovaccine exhibited potent antitumor efficacy, as evidenced by marked tumor suppression and increased tumor infiltration of CD8^+^ T cells, while maintaining favorable biosafety throughout the treatment period. Taken together, this combinatorial strategy enables spatiotemporal control of immune modulation, offering a versatile platform for precision cancer photodynamic immunotherapy.

## CONFLICT OF INTEREST STATEMENT

The authors declare no conflicts of interest.

## ETHICS STATEMENT

All animal procedures were performed in accordance with the guidelines for Care and Use of Laboratory Animals of Dalian Medical University and approved by the Dalian University of Technology Animal Care and Use Committee.

## Supporting information

Supporting Information S1

## Data Availability

The data that support the findings of this study are available from the corresponding author upon reasonable request.
