# Supplementary material for: A photoactivatable tumor‐targeting in situ nanovaccine for large‐volume tumor therapy
Source: Smart Mol. 2025 Aug 19;3(3):e70014. doi: 10.1002/smo2.70014 (PMC12483135; doi:10.1002/smo2.70014)
Supplement: Supplementary file 1 — Supporting Information S1 [file SMO2-3-e70014-s001.pdf]

Smart Molecules Open Access

WILEY

Supporting Information

A Photoactivatable Tumor-targeting In Situ Nanovaccine for Large-volume Tumor Therapy

*Junying Ding[1], Xueze Zhao[2], Saran Long[1], Wen Sun[1,3], Jianjun Du[1,3], Jiangli Fan[1,3] and Xiaojun Peng[1,4]**

[1]State Key Laboratory of Fine Chemicals, Frontiers Science Center for Smart Materials Oriented Chemical Engineering, Dalian University of Technology, Dalian (China).

[2]Department of Chemistry, The University of Hong Kong, Hong Kong (China).

[3]Ningbo Institute of Dalian University of Technology, Ningbo (China).

[4]State Key Laboratory of Fine Chemicals, College of Materials Science and Engineering, Shenzhen University, Shenzhen (China).

EXPERIMENTAL PROCEDURES

Materials and instrumentation

All manipulations with organic compounds were degassed performed under a dry argon atmosphere. The general chemicals used in this study were analytically pure and purchased from Energy Chemical Co. or MedChemExpress. Hoechst 33324 was purchased from Titan Technology Co., Ltd (Shanghai, China). 3-(4,5-dimethylthiazol-2-yl)-2,5-diphenyltetrazolium bromide (MTT), Alexa Fluor 488-labeled goat anti-rabbit IgG(H+L), Reactive Oxygen Species Assay Kit, ATP assay kit and Calcein/PI Cell Viability/Cytotoxicity Assay Kit were purchased from Beyotime Biotechnology Co., Ltd. (Shanghai, China). Calreticulin polyclonal antibody and HMGB1 polyclonal antibody were purchased from Proteintech Group, Inc (Wuhan, China). Flow cytometry antibodies were purchased from Biolegend. Milli-Q water was supplied by Milli-Q Plus System (Millipore Corporation, United States).

^{1}H NMR and ^{13}C NMR spectra were detected by a Bruker Avance 500M or 400M NMR Spectroscopy (Bruker). Mass spectrometry was carried out using Q-TOF LC/MS (Agilent). Absorption spectra were measured on a CARY 60 UV-Vis spectrophotometer (Agilent). Fluorescence spectra were obtained with an Agilent Cary Eclipse fluorescence spectrophotometer (Agilent). Confocal laser scanning microscope (CLSM) images were performed on an Olympus FV3000 CLSM (Olympus). Small animals' fluorescence imaging was carried out by the NightOWL II LB983 living imaging system (Berthold Technologies). The size distribution was determined using dynamic light scattering (DLS) measurement (Zetasizer Nano, Malvern Instrument). The morphology was examined using transmission electron microscope TEM (HT7800).

Cell culture.

Dulbecco's Modified Essential Medium (DMEM) and phosphate-buffer saline (PBS) buffer with pH = 7.4 are commercial product of Solarbio Science & Technology Co., Ltd (Beijing, China). Murine 4T1 breast cancer cells were maintained in DMEM with 10% FBS and incubated at 37 °C under a humidified atmosphere containing 5% of CO_2. All experiments were performed in the logarithmic phase of cells growth.

Animals

Female Balb/c mice (6-7 weeks, 18-20 g) were obtained from the Liaoning Changsheng biotechnology co., Ltd. (Liaoning, China).

Preparation and Characterization of Lipo-D8-6

Lipo-D8-6 nanoparticles was synthesized using a thin-film hydration method. Briefly, a mixture of soy bean lecithin, cholesterol, DSPE-mPEG, and DSPE-PEG-cRGD was dissolved in 20 mL chloroform at the ratio of 62:33:4:1 within a single-neck flask. To ensure complete dissolution

of the lipid components, the mixture was subjected to ultrasonic. Then, a chloroform solution containing D8 and Ce6 was introduced into the lipid mixture, and the organic solvents were removed using rotary evaporation to form a film layer, which was further dried in vacuo. The dried mixture was then re-dissolved by 20 mL PBS, and the obtained solution was cycled under high-pressure homogenizer for 30 times and quickly filtered with syringe filter. Finally, the product was stored at 4 °C.

The size distribution of Lipo-D8-6 was assessed using dynamic light scattering (DLS) measurement. The morphology of Lipo-D8-6 was examined using TEM. To investigate the serum stability, Lipo-D8-6 was incubated in 10% FBS and the size monitored for 24 h.

Photoactivatable Drug Release

The light-triggered drug release behavior of nanoparticles was investigated using high performance liquid chromatography. Lipo-D8-6 working solution (500 μL) was subjected to uniform irradiation using a 660 nm light (50 mW/cm^2). The drug release efficiency was determined through analysis of chromatographic peak areas at fixed time. And mass spectrometry analysis was performed on the chromatographic peak before and after light irradiation.

Cellular Uptake

To assess the uptake efficiency of Lipo-D8-6, 4T1 cells were cultured in glass-bottom culture dishes overnight at 37 °C. Following three times wash with PBS, cells were incubated with culture medium containing 5 μM Lipo-D8-6. The process of cellular uptake was monitored by confocal laser scanning microscopy every two hours.

Detection of cellular ROS generation

4T1 cells were seeded in glass-bottom culture dishes and cultured overnight at 37°C.

Afterwards, cells were incubated with 5 μM Lipo-D8-6 for 4 h. Then, cells were incubated with 10 μM DCFH-DA for 30 min and cells were washed with PBS. Cells in the light treated group were exposed to 660 nm light. Then, the intracellular ROS generation was analyzed using confocal laser scanning microscopy.

Cell viability assay

Cell viability was assessed using the MTT assay. Briefly, 4T1 cells were cultured in 96 well plates overnight at 37°C. Then, cells were incubated with culture medium containing varying concentrations of Lipo-D8-6. After incubation for 4 h, cells in the light treated group were exposed to 660 nm light irradiation (12 J/cm^2), followed by incubation for 12 h at 37°C. Subsequently, MTT (5 mg/mL) solution was added to each well, and incubated for another 4 h. Afterwards, the culture medium was replaced with 150 μL of dimethyl sulfoxide, and the absorbance at 490 nm was measured using a microplate reader and the cell viability was calculated by the following equation:

$$\text{Cell viability (\%)} = \frac{\text{OD}_{PS} - \text{OD}_{blank\ control}}{\text{OD}_{control} - \text{OD}_{blank\ control}} \times 100\%$$

To evaluate the dark toxicity, the same procedure as the phototoxicity evaluation was followed, with the exception of the light irradiation step.

Cell viability/cytotoxicity assay

4T1 cells were cultured in glass-bottom culture dishes overnight at 37 °C, then incubated with Lipo-D8-6 for 4 h and cells in the light treated group were exposed to 660 nm light irradiation. Then, cells stained with calcein acetoxymethyl ester (Calcein-AM) and propidium iodide (PI) for 30 min and was analyzed using confocal laser scanning microscopy.

Analysis of the ICD Effect.

The capability of Lipo-D8-6 to induce ICD, including CRT exposure and the release of HMGB1 and ATP, was measured.

CRT exposure and HMGB1 release were measured by CLSM. 4T1 cells were seeded in glass-bottom culture dishes and cultured overnight at 37°C. Next day, cells were cultured with Lipo-D8-6 for 4 h and cells in the light treated group were exposed to 660 nm light irradiation. After incubation for another 2 h, the cells were harvested, fixed with 4% paraformaldehyde, permeabilized with Triton X-100 and blocked with blocking solution. Then, cells were incubated with CRT antibody or HMGB1 antibody at 4°C overnight, followed by incubation with Alexa Fluor 488-conjugated secondary antibody at room temperature for 2 h. Finally, cells were stained with Hoechst 33342 and observed by CLSM.

The extracellular ATP secretion was analyzed by using a commercial ATP assay kit. Briefly, the culture medium of 4T1 cells treated with various formulations was collected to measure its ATP content according to the manufacturer's instructions.

Measurement of BMDC maturation *in vitro*

BMDCs derived from C57BL/6 mice. The effect of Lipo-D8-6 on BMDCs maturation was measured using a transwell system. Specifically, BMDCs were cultured in the lower compartments and the upper compartments contained 4T1 cells subjected to different treatments. Then, cells were collected after 24 h, and mature BMDCs-related markers ($CD11c^+$ $CD80^+$ $CD86^+$) were analyzed using flow cytometry.

In Vivo Imaging

Biodistribution of the nanovaccine was assessed by intravenously injecting Lipo-D8-6 into tumor-bearing Balb/c mice. Then the fluorescence intensity was measured by the *in vivo* imaging system at different times postinjection. The major organs and tumor were harvested for investigating biodistribution of the nanovaccine *ex vivo*.

Antitumor activity of Lipo-D8-6

Large-volume orthotopic breast tumor-bearing mice were randomly divided into 5 groups after the tumor volume reached 300 mm^3 (n = 3), including PBS Dark、PBS Light、Lipo-D8-6 Dark、Lipo-6 Light and Lipo-D8-6 Light. The mice were intravenously administered with various formulations at an equal dosage of Ce6 (3.5 mg/kg). The light groups were exposed to 650 nm light irradiation (90 J/cm^2). The tumor growth and body weight change were monitored every 2 days. The tumor volume was calculated by follow formula: (L, the longest dimension; W, the shortest dimension)

$$V=L*W*W/2$$

The tumors were harvested at the end of antitumor studies, fixed in 4% formalin solution, dehydrated and subjected to immunofluorescence examination and H&E staining.

The major organs (e.g., hearts, livers, spleens, lungs and kidneys) were harvested at the end of antitumor studies, fixed in 4% formalin solution, dehydrated and subjected to H&E staining.

Statistical Analysis

Data were expressed as the mean ± standard deviation. One-way ANOVA test with a Tukey post hoc test was used to evaluate the statistical significance. *$p < 0.05$, **$p < 0.01$, ***$p < 0.001$, ****$p < 0.0001$).

Synthesis of compound 1

2,2'-(Propane-2,2-diylbis(sulfanediyl))diethanol (0.51 mmol) and N,N'-Disuccinimidyl carbonate (2.04 mmol) were dissolved in 10 mL anhydrous acetonitrile, followed by the addition of anhydrous pyridine (2.04 mmol). The reaction mixture was stirred overnight under the nitrogen atmosphere. The reaction was monitored using a TLC. The crude product was subsequently purified by silica gel column chromatography using dichloromethane and

methanol (100:1) to obtain compound 1 (yield, 68%). ^{1}H NMR (400 MHz, $CDCl_3$) δ (ppm): 4.47 (t, J = 7.0 Hz, 4H), 3.00 (t, J = 7.0 Hz, 4H), 2.87 – 2.83 (m, 8H), 1.63 (s, 6H)。

Synthesis of compound D8

Under the nitrogen atmosphere, a mixture of compound 1 (0.21 mmol) and R848 (0.52 mmol) was dissolved in 20 mL anhydrous dichloromethane. After addition of anhydrous triethylamine (0.52 mmol), the reaction mixture was stirred overnight at room temperature. The reaction was monitored using a TLC. The crude product was subsequently purified by silica gel column chromatography using dichloromethane and methanol (50:1) to obtain the compound D8 (yield, 42%). ^{1}H NMR (500 MHz, $CDCl_3$) δ (ppm): 8.13 (dd, J = 12.8, 8.4 Hz, 4H), 7.58 (t, J = 7.4 Hz, 2H), 7.46 (t, J = 7.6 Hz, 2H), 4.86 (s, 4H), 4.71 (s, 4H), 4.46 (t, J = 6.7 Hz, 4H), 3.62 (q, J = 7.0 Hz, 4H), 3.04 (t, J = 6.7 Hz, 4H), 1.67 (s, 6H), 1.32 – 1.20 (m, 18H). ^{13}C NMR (151 MHz, $CDCl_3$) δ (ppm): 151.20, 144.44, 135.77, 127.69, 127.42, 124.65, 120.18, 116.54, 71.39, 66.65, 65.01, 64.36, 63.72, 56.57, 56.41, 53.43, 31.04, 29.47, 27.86, 27.22, 14.92, 14.13. HRMS ($C_{43}H_{56}N_8O_8S_2$) m/z: $[M+H]^+$ calcd 877.3736, found 877.3732.

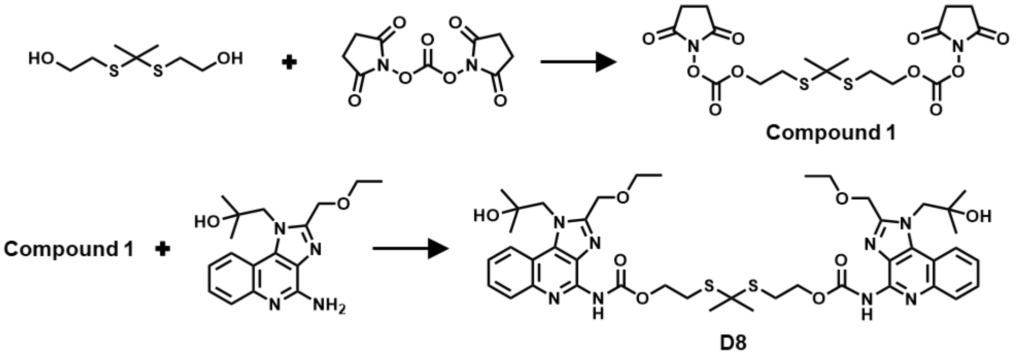

Figure S1. Synthesis routes of D8.

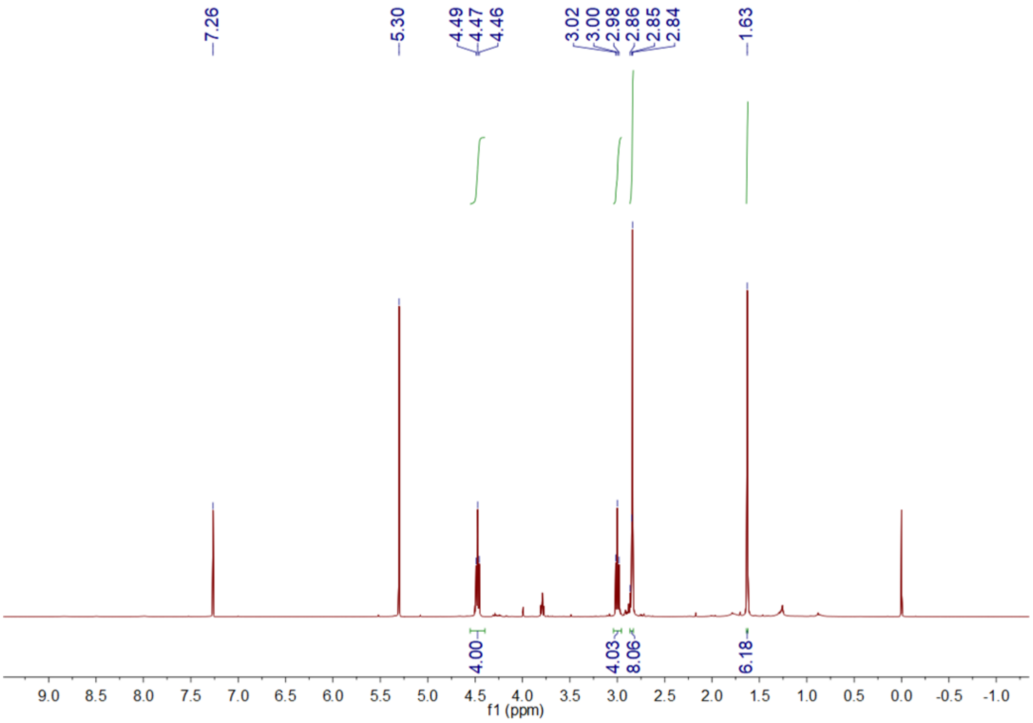

Figure S2. ^{1}H NMR spectrum of compound 1.

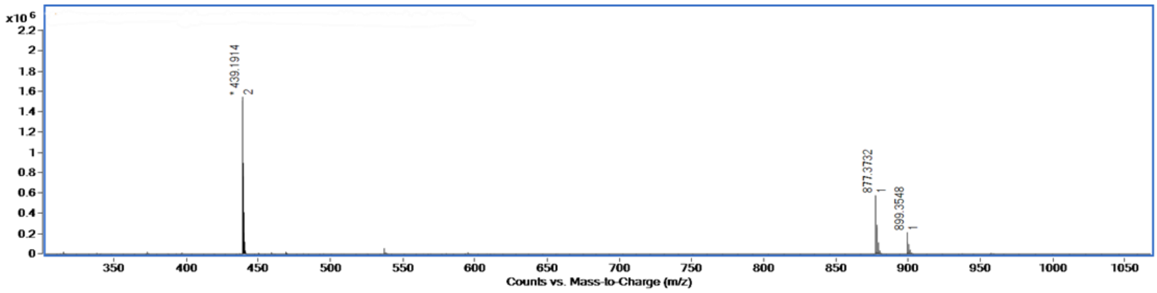

Figure S3. Mass spectrum of compound D8.

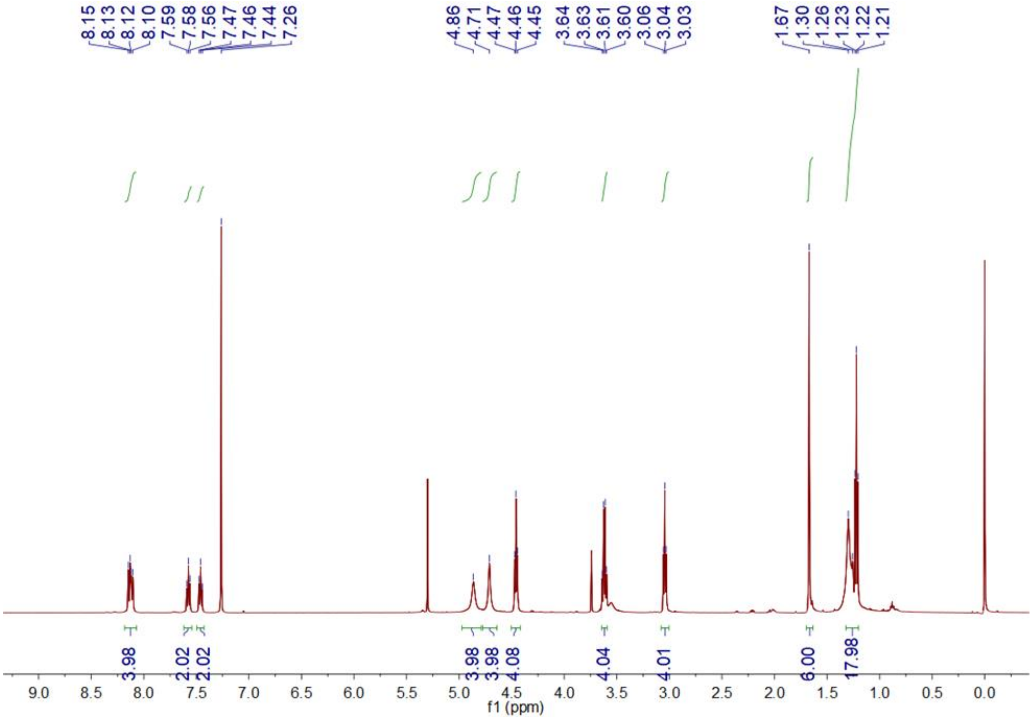

Figure S4. ^{1}H NMR spectrum of compound D8.

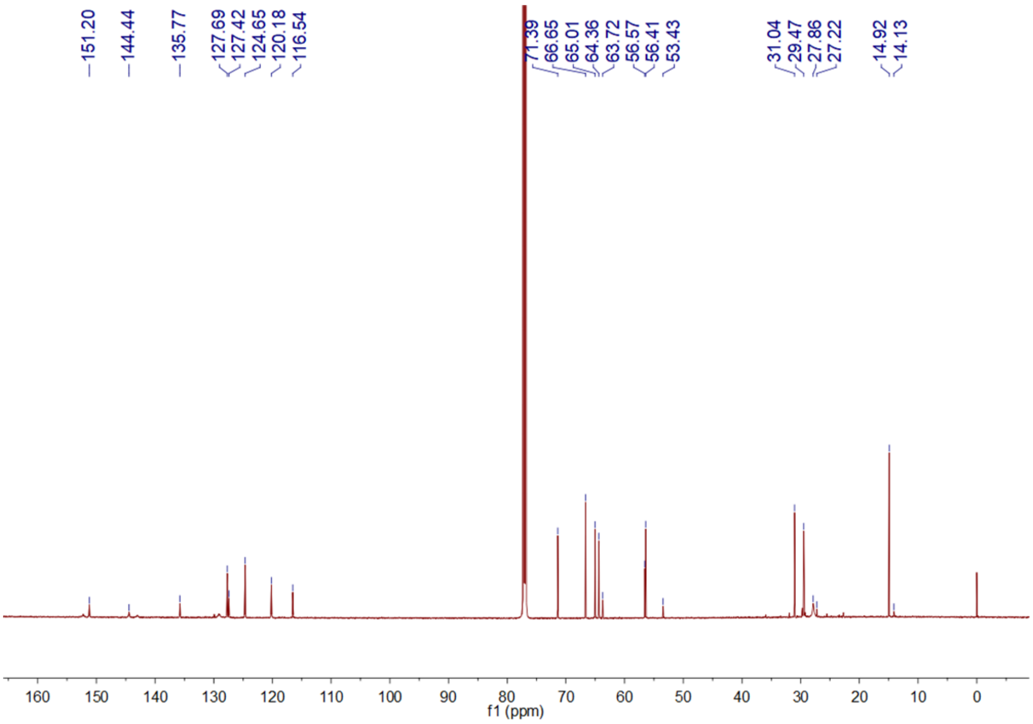

Figure S5. ^{13}C NMR spectrum of compound D8.

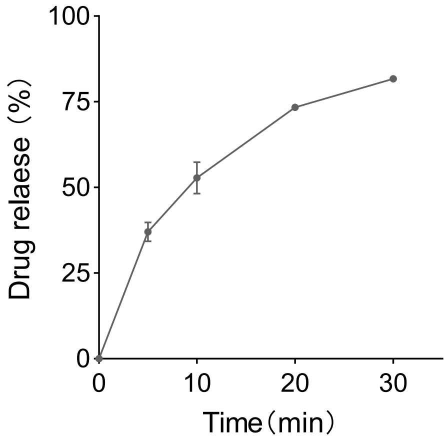

Figure S6. The R848 release profile of Lipo-D8-6 with 660 nm (50 mW/cm²) light irradiation.

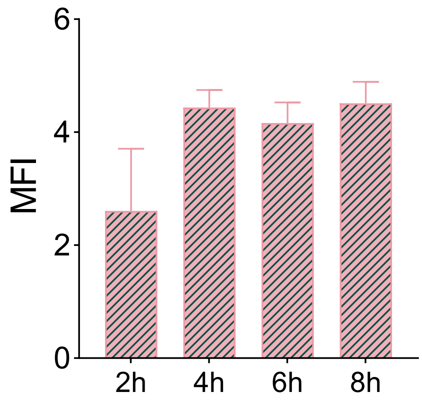

Figure S7. MFI of cell uptake of Lipo-D8-6

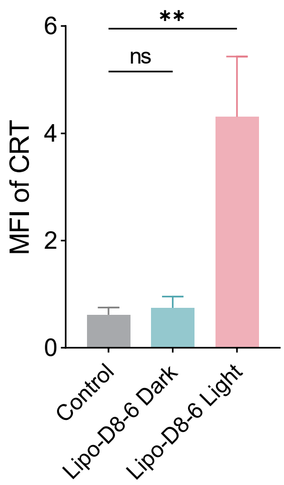

Figure S8. MFI of CRT in 4T1 cells after different treatments

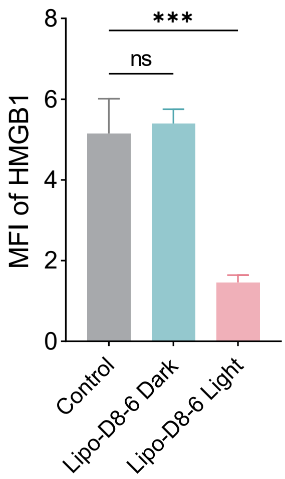

Figure S9. MFI of HMGB1 in 4T1 cells after different treatments

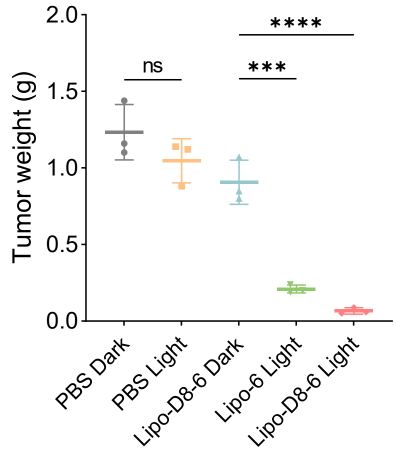

Figure S10. Tumor weight of excised tumors in each group.

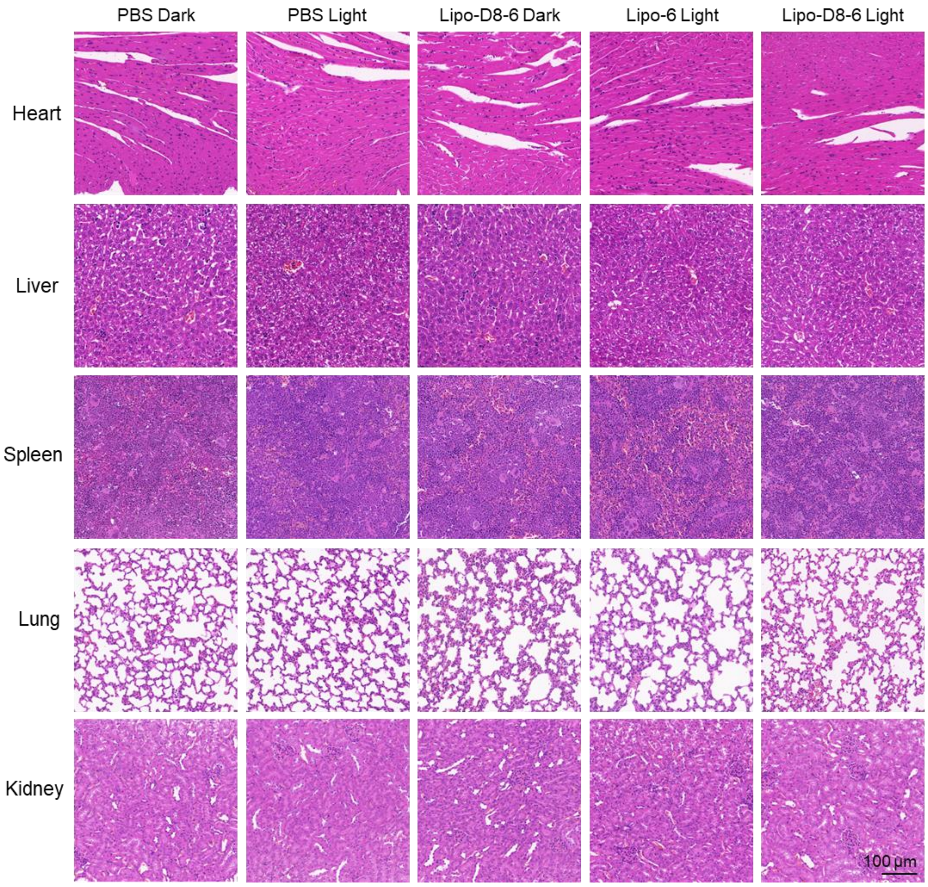

Figure S11. H&E staining of the major organs from tumor-bearing mice after different treatments.
